# β-catenin-IRP2-primed iron availability to mitochondrial metabolism is druggable for active β-catenin-mediated cancer

**DOI:** 10.1186/s12967-023-03914-0

**Published:** 2023-01-26

**Authors:** Yuting Wu, Shuhui Yang, Luyang Han, Kezhuo Shang, Baohui Zhang, Xiaochen Gai, Weiwei Deng, Fangming Liu, Hongbing Zhang

**Affiliations:** 1 State Key Laboratory of Medical Molecular Biology, Haihe Laboratory of Cell Ecosystem, Department of Physiology, Institute of Basic Medical Sciences and School of Basic Medicine, Chinese Academy of Medical Sciences and Peking Union Medical College, 5 Dong Dan San Tiao, Beijing, China; 2 Department of Physiology, School of Life Science, China Medical University, Shenyang, China

**Keywords:** β-catenin, IRP2, Labile iron pool, Iron chelator, Mitochondrial function

## Abstract

**Background:**

Although β-catenin signaling cascade is frequently altered in human cancers, targeting this pathway has not been approved for cancer treatment.

**Methods:**

High-throughput screening of an FDA-approved drug library was conducted to identify therapeutics that selectively inhibited the cells with activated β-catenin. Efficacy of iron chelator and mitochondrial inhibitor was evaluated for suppression of cell proliferation and tumorigenesis. Cellular chelatable iron levels were measured to gain insight into the potential vulnerability of β-catenin-activated cells to iron deprivation. Extracellular flux analysis of mitochondrial function was conducted to evaluate the downstream events of iron deprivation. Chromatin immunoprecipitation, real-time quantitative PCR and immunoblotting were performed to identify β-catenin targets. Depletion of iron-regulatory protein 2 (IRP2), a key regulator of cellular iron homeostasis, was carried out to elucidate its significance in β-catenin-activated cells. Online databases were analyzed for correlation between β-catenin activity and IRP2-TfR1 axis in human cancers.

**Results:**

Iron chelators were identified as selective inhibitors against β-catenin-activated cells. Deferoxamine mesylate, an iron chelator, preferentially repressed β-catenin-activated cell proliferation and tumor formation in mice. Mechanically, β-catenin stimulated the transcription of IRP2 to increase labile iron level. Depletion of IRP2-sequered iron impaired β-catenin-invigorated mitochondrial function. Moreover, mitochondrial inhibitor S-Gboxin selectively reduced β-catenin-associated cell viability and tumor formation.

**Conclusions:**

β-catenin/IRP2/iron stimulation of mitochondrial energetics is targetable vulnerability of β-catenin-potentiated cancer.

**Supplementary Information:**

The online version contains supplementary material available at 10.1186/s12967-023-03914-0.

## Background

WNT/β-catenin pathway plays critical roles in cellular physiology and pathology. Upon WNT activation, β-catenin escapes from its degradation complex and translocates to nucleus. Nuclear β-catenin interacts with transcription factors and chromatin modifiers such as T cell factor (TCF) and cyclic adenosine monophosphate response element binding protein-binding protein (CBP) to stimulate target gene expression [1]. By promoting the expression of cyclin D1, fibroblast growth factor, glutamate-ammonia ligase (GLUL), peroxisome proliferator activated receptor alpha (PPARα), and telomerase reverse transcriptase, β-catenin regulates cell proliferation, survival, metabolism and genetic stability [2–6]. Activation of WNT/β-catenin pathway is prevalent in human cancers including hepatic, colorectal, endometrial, and pancreatic cancers [7–10]. Active mutations of β-catenin, encoded by catenin beta 1 (*CTNNB1)*, or loss-of-function mutations of its destruction complex, such as adenomatous polyposis coli (*APC*), *AXIN1* and *AXIN2*, lead to β-catenin stabilization and eventually unrestrained transcriptional activity [11–13]. According to cBioPortal database, *APC* is the top 1 mutated tumor suppressor gene in colon adenocarcinoma (COAD, 68.6% mutation rate) and *CTNNB1* (30.6% mutation rate) is the most frequently mutated proto-oncogene in hepatocellular carcinoma (HCC) [14, 15]. In uterine corpus endometrial carcinoma (UCEC), *CTNNB1* is also highly altered (34.6% mutation rate) [14, 15]. The importance of β-catenin in carcinogenesis has provided rationale for the study of its oncogenic mechanisms and the development of therapeutics that selectively inhibits β-catenin-activated tumors.

Various compounds are investigated to intervene WNT/β-catenin pathway for cancer treatment. A β-catenin degrader, proteolysis targeting chimeras, suppress WNT-dependent colorectal cancer (CRC) in mice [16].Targeting β-catenin-TCF transcription complex with CCT036477, iCRT3, iCRT5, iCRT14 or PKF118-310 preferentially represses the survival of malignant cells in preclinical studies [17–20]. Pri-724, a first-in-class antagonist that blocks β-catenin/CBP interaction, is being studied in clinical trials to treat CRC, pancreatic cancer, acute myeloid leukemia and chronic myelogenous leukemia [21, 22]. Besides, inhibitions of β-catenin downstream AKT Serine/Threonine Kinase 2 (AKT2)-stimulated pyrimidine synthesis axis or PPARα-driven fatty acid oxidation (FAO) blocked β-catenin-activated tumor formation [5, 23]. Despite these substantial efforts, none of them have been approved for clinical application.

Given the escalating cost, slow pace, and high attrition rates of translating new drugs into clinical practice, repurposing of old drugs is an attractive proposition [24]. Taking an unbiased screening of compound library of approved drugs, we identified iron chelators as selective inhibitors of β-catenin-activated cell proliferation and tumorigenesis. β-catenin-stimulated IRP2 (iron regulating protein 2)-iron homeostasis was essential for mitochondrial function. Targeting β-catenin-IRP2-iron-mitochondria cascade is an appealing therapeutic strategy for β-catenin-activated tumors.

## Methods

### Reagents

Deferoxamine mesylate (DFO, #D9533), ferric ammonium citrate (FAC, #F3388), and insulin (#I9278) were purchased from Sigma-Aldrich (St. Louise, MO, USA). Deferasirox (DFX, #S1712), oligomycin A (#S1478), hydrocortisone hemisuccinate (#S5972), and pri-724 (#S8968) were purchased from Selleck Chemicals (Houston, TX, USA). VLX600 (#T8500) was purchased from Target Molecule (Boston, MA, USA). S-Gboxin was purchased from BiochemPartner Biotech (Shanghai, China). Lipofectamine 2000 (#11668019), TRIzol (#15596018), trypsin–EDTA (#25200056), DMEM (#11995–065), RPMI-1640 (#72400047), fetal bovine serum (FBS, #10100147C), nonessential amino acid solution (NEAA, #11140050), penicillin G and streptomycin (#15140163, P/S), glutamax (#35050061), and sodium pyruvate (#11360070) were purchased from Thermo Fisher (Carlsbad, CA, USA). Horse serum was purchased from Procell (#164215, Wuhan, Hubei, China). Puromycin (#P8230), 0.1% crystal violet (#G1063), and polybrene (#H8761) were purchased from Solarbio (Beijing, China). 4% paraformaldehyde (PFA) was purchased from Servicebio (#G1101, Wuhan, Hubei, China). Cell counting kit-8 (CCK8) was purchased from Yeasen Biotech (#40203ES60, Shanghai, China). *β-catenin*^*mut*^ plasmid was purchased from Addgene (#24204, Watertown, MA, USA).

### High-throughput drug screening

A panel of 1088 compounds from an FDA-approved drug library (Selleck chemicals) was screened for drugs which preferentially target β-catenin-activated cells. In brief, wildtype (WT) or *β-catenin*^*Δ(ex3)/*+^ mouse embryonic fibroblasts (MEFs) were plated in 384-well plates with 500 cells per well and treated with DMSO or 10 μM compounds using Echo^®^ 550 Liquid Handler (Beckman counter, Brea, CA, USA). After 72 h incubation, cell viability was determined with CCK8 assay using EnVision Multilabel Plate Reader (PerkinElmer, Waltham, MA, USA). The high-throughput screening was repeated for 3 times. Inhibition rate was normalized against DMSO treatment.

### Cell lines

WT, *β-catenin*^*Δ(ex3)/*+^ MEFs and MHCC97H were previously reported [23]. Cells were maintained at 37 °C incubator with 5% CO_2_. The supplier and culture medium of hepatic cell lines were shown as follow:

**Table:** 

Cell lines	Supplier	Culture medium
HepaRG	Thermo Fisher	DMEM + 10% FBS + 1% NEAA + 1% P/S + 1% glutamax + 5 μg/ml insulin + 50 μM hydrocortisone hemisuccinate
293FT	Abclonal (Wuhan, Hubei, China)	DMEM + 10% FBS + 1% P/S
CCC-HEL-1	China Infrastructure of Cell Line Resource (Beijing, China)	DMEM + 20% FBS + 1% P/S
NCTC1469	China Infrastructure of Cell Line Resource	DMEM + 10% horse Serum + 1% P/S
HepG2	China Infrastructure of Cell Line Resource	DMEM + 10% FBS + 1% P/S
SNU886	Cobioer biosciences (Nanjing, Jiangsu, China)	RPMI-1640 + 10% FBS + 1% P/S
HuH7	Procell	RPMI-1640 + 10% FBS + 1% P/S
Hepa 1–6	Procell	DMEM + 10% FBS + 1 mM Sodium Pyruvate + 1% P/S
SNU182	Procell	RPMI-1640 + 20% FBS + 1% P/S
SNU398	Sagen tech (Guangzhou, Guangdong, China)	DMEM + 10% FBS + 1% P/S
HCC-LM3	China Infrastructure of Cell Line Resource	DMEM + 10% FBS + 1% P/S

### siRNA and shRNA transfections

Cells were transfected with *β-catenin* siRNA or *Irp2* shRNA (Tsingke Biotech, Beijing, China) using lipofectamine 2000 according to the manufacturer’s instructions. Sequences were followed:

si-Human *β-catenin*-1: 5′-TTGTTATCAGAGGACTAAAT-3′.

si-Human *β-catenin*-2: 5′-TCTAACCTCACTTGCAATAAT-3′.

si-Mouse *Tcf7l2*-1: 5′-CCTTAGCGTAAGCATCTTATA-3′.

si-Mouse *Tcf7l2*-2: 5′-GCTCCGAAAGTTTCCGAGATA-3′.

si-Human *TCF7L2*-1: 5′-GCGGGATAACTATGGAAAGAA-3′.

si-Human *TCF7L2*-2: 5′-CGAACCTATCTCCAGATGAAA-3′.

sh-*Irp2*-1:5′-CCGGCCTGCCAGTTACTCTTACTTTCTCGAGAAAGTAAGAGTAACTGGCAGGTTTTTT-3′.

sh-*Irp2*-2:5′-CCGGCGATAGAACTACCATAGCAAACTCGAGTTTGCTATGGTAGTTCTATCGTTTTTT-3′.

### Cell viability and colony formation

For cell viability, MEFs and hepatic cell lines were seeded in 96-well plates at a density of 3000 to 6000 cells per well. Cells were treated with different concentrations of compounds for indicated times and then incubated with CCK8 for 4 h at 37 °C. The absorbance at 450 nm wavelength was measured by microplate reader (Agilent, Santa Clara, CA, USA). For colony formation, 200 cells were plated in six-well plates. Cells were fixed in 4% PFA for 15 min and then stained with 0.1% crystal violet for half-hour.

### Extracellular flux analysis of oxygen consumption rate (OCR) and ATP production rate

OCR and ATP analysis was performed using Seahorse Xfe 24 flux analyzer (Agilent). 10,000 cells were seeded in 24-well microplates. Before measurement, cell medium was replaced by XF Assay Medium supplemented with pyruvate, glucose, and glutamine (pH 7.4). Measurement was done at baseline and sequential injections of 1 μM oligomycin, 2 μM FCCP and 0.5 μM rot/antimycin A for OCR, or 1.5 μM oligomycin and 0.5 μM rot/antimycin A for ATP respectively. Data were represented as normalization to cell numbers.

### Labile iron pool (LIP) measurement

Intracellular labile iron pool analysis was performed with Phen Green™ SK (PGSK) fluorescent probe (#P14313, Thermo Fisher). For flow cytometry analysis, cells were harvested and washed with PBS after incubating with 2 μM PGSK for 15 min. Samples were then analyzed on BD Accuri C6 or C6 plus (Franklin Lakes, NJ, USA). For fluorescent images, cells incubated with 2 μM PGSK were fixed in 4% PFA for 15 min and washed with PBS for 3 times before observation by LSM780 (Zeiss, Oberkochen, Germany).

### Real-time quantitative PCR

Total RNA was extracted from cells with TRIzol reagent. cDNA was generated using Hifair® II 1st Strand cDNA Synthesis Kit (gDNA digester plus) (#11121ES60, Yeasen) according to the manufacturer’s protocol. Realtime-PCR was performed using SYBR High-Sensitivity qPCR SuperMix (#abs60086, Absin Biosciences, Shanghai, China). β*-*actin transcripts were used to normalize the expression of each gene. The primer sequences were as follows:

Mouse *Irp2*-F: 5′-TTCTGCCTTACTCAATACGGGT-3′.

Mouse *Irp2*-R: 5′-AGGGCACTTCAACATTGCTCT-3′.

Mouse *Glul*-F: 5′-TGAACAAAGGCATCAAGCAAATG-3′.

Mouse *Glul*-R: 5′-CAGTCCAGGGTACGGGTCTT-3′.

Mouse *β-actin*-F: 5′-AGAGGGAAATCGTGCGTGAC-3′.

Mouse *β-actin*-R: 5′-CAATAGTGATGACCTGGCCGT-3′.

Human *IRP2*-F: 5′-GGAATTCCATATGATACAGAATGCACCAAAT-3′.

Human *IRP2*-R: 5′-CGGGATCCTCATGTTTCAGGTTCAGCCAC-3′.

Human *β-actin-F:* 5′-*CCTGGCACCCAGCACAAT*-3′.

Human *β-actin-R:* 5′-GCCGATCCACACGGAGTACT-3′.

### Immunoblotting

Cell proteins were extracted from SDS loading buffer with Protease and Phosphatase Inhibitor Cocktail (EDTA-Free) (#P002, New cell & molecular, Suzhou, Jiangsu, China). Immunoblotting was conducted as previously described [23]. Membranes were first incubated with primary antibodies against β-catenin (#9587, Cell Signaling Technology, Danvers, MA, USA), Cyclin D1 (#A19038, Abclonal), IRP2 (#A6382, Abclonal), IRP1 (#A7867, Abclonal), TfR1 (#A5865, Abclonal), TCF7L2 (#A20770, Abclonal), FBXL5 (#A5602, Abclonal) or GAPDH (#AC002, Abclonal) overnight at 4 °C, and then incubated with secondary IRDye 680RD goat anti-rabbit (#68071, LI-COR Biosciences, Lincoln, USA) or IRDye 800 CW goat anti-mouse (#32210, LI-COR Biosciences) antibodies for 2 h at room temperature. Protein bands were detected by LI-COR Odyssey Infrared Scanner.

### Chromatin immunoprecipitation (ChIP)

Β-catenin ChIP-seq data analysis was conducted using our published datasets (GSE165853). For the bioinformatic analysis of ChIP-seq data, Bowtie2 was used for alignment, MACS2 with FDR < 0.01 was for enriched peaks identification, and ChIPseeker was for peaks annotation. Peaks enrichment distribution analysis was performed by Deeptools as previously described [23]. ChIP assays were performed for *β-catenin*^*Δ(ex3)/*+^ MEFs using SimpleChIP Plus Enzymatic Chromatin IP Kit (Magnetic Beads) (#9005, Cell Signaling Technology). Briefly, cells were seeded on 15 cm plates, cross-linked with 1% formaldehyde, and stopped with glycine. Cells were harvested with lysis buffer containing protease inhibitor complex. The lysates were digested by micrococcal nuclease and then sonicated to shear the DNA. Chromatins were immunoprecipitated with 20 μl anti-β-catenin (106 μg/ml), 10 μl anti-Histone H3 (269 μg/ml, positive control) or 2 μl anti-IgG (1 mg/ml) antibody overnight at 4 °C on a rotating wheel according to manufacturer’s instructions. The ChIP-enriched DNA samples were purified and then amplified by real-time PCR using primers as followed, and data were expressed as a percentage of the total input chromatin by the formula: percent Input = 2% × 2^(C[T] 2%Input Sample − C[T] IP Sample)^, C[T] = Threshold cycle of PCR reaction.

Site1-F: 5′-GTGAGCGCTGTGATGCAATA-3′.

Site1-R: 5′-CCTTGGTGCTGTTGGTATCTG-3′.

Site2-F: 5′-TAGCCAACGGTGTACAGGAG-3′.

Site2-R: 5′-TGAGTCTGGATTTGCGATGC-3′.

Site3-F: 5′-GCATCGCAAATCCAGACTCA-3′.

Site3-R: 5′-TTGATCCACTGGCTTCCTGAC-3′.

Site4-F: 5′-GATGCCTGTGGTAACAAGCG-3′.

Site4-R: 5′-ATTGGCTCCAGCTATCCGTT-3′.

Site5-F: 5′-GCAAGAGCTGGGGTATTAGCA-3′.

Site5-R: 5′-GGAGTTGTCTGGTTTGCCCT-3′.

### Subcutaneous tumor formation

All animal studies were performed following the protocols approved by the Animal Center of the Institute of Basic Medical Sciences, Chinese Academy of Medical Sciences and Peking Union Medical College (ethical code: ACUC-A02-2022-057). Female BALB/c nude mice (4–6 weeks) were purchased from HFK Bio-Technology (Beijing, China). 1 × 10^6^ *β-catenin*^*Δ(ex3)/*+^ MEFs suspended in 100 μL PBS or 2 × 10^6^ HuH7 in 100 μL PBS containing 50% Matrigel (#354234, BD Biosciences, San Jose, CA, USA) were subcutaneously inoculated in the right dorsal flank of nude mice. When tumor volumes reached 100 mm^3^, mice were randomly assigned to different groups. The treatment group was intraperitoneally injected with 10 mg/kg S-Gboxin, or 400 mg/kg DFO every other day and control group was injected with equal volume of vehicle. Tumor volumes and body weights were measured every other day and tumor volumes were calculated by the formula: volume = length × width^2^/2.

### Database analysis

For gene expression correlation between *CTNNB1* and *IRP2* or *TFRC*, cell line analysis was conducted with Dependency Map (DepMap; https://depmap.org/portal/interactive), and human sample analysis was performed using Gene Expression Profiling Interactive Analysis 2.0 (GEPIA2; http://gepia.cancer-pku.cn/detail.php) [25]. UALCAN was analyzed for protein expression (http://ualcan.path.uab.edu/analysis-prot.html) [26]. JASPAR database was analyzed for TCF7L2 binding motif at *IRP2* promoter (https://jaspar.genereg.net) [27].

### Statistical analysis

Data were expressed as mean ± SD. All data were independently repeated for at least 3 times. Statistical analysis was carried out using a two-tailed unpaired t-test with GraphPad Prism software. *P* < 0.05 was considered as statistically significant.

## Results

### β-catenin-activated cells are more susceptible to iron chelators

**Fig. 1:**
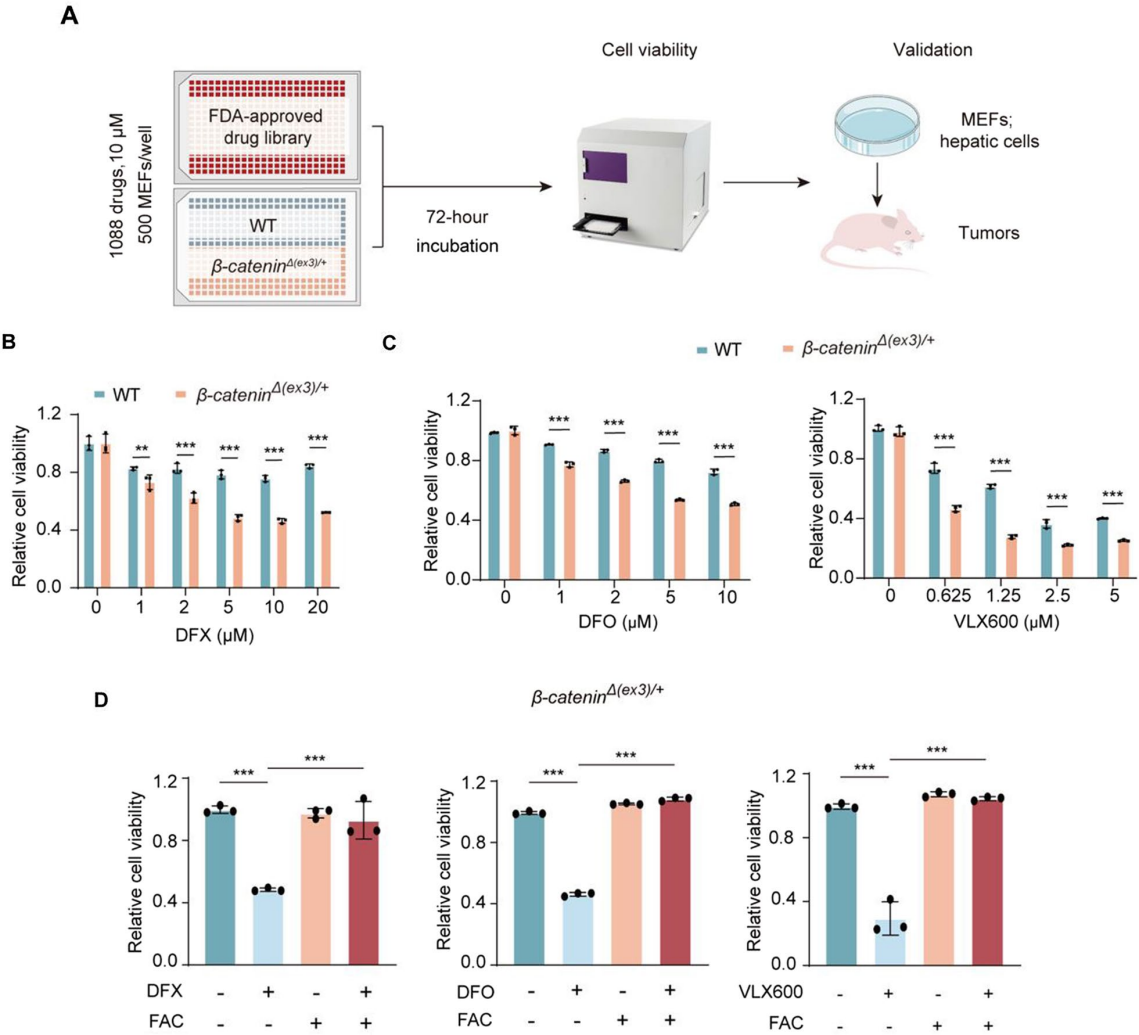
Identification of iron chelators as selective inhibitors for β-catenin-activated cells. **A**, Schematic illustration of drug screening and validation. Wildtype (WT) and *β-catenin*^*Δ(ex3)/*+^ MEFs were treated with a chemical library containing 1088 FDA-approved drugs at a concentration of 10 μM for 72 h. Cell viability was measured. Candidate hits were verified in MEFs, hepatic cells and subcutaneous tumors of mice. **B**–**D,** Cell viability. WT and *β-catenin*^*Δ(ex3)/*+^ MEFs were treated with DFX (**B**), DFO (**C**, left) and VLX600 (**C**, right) at different concentrations for 48 h. *β-catenin*^*Δ(ex3)/*+^ MEFs were treated with DFX, DFO or VLX600 in the presence or absence of FAC for 48 h (**D**);* n* = 3. Data were shown as mean ± SD and analysis was performed using *t* test. **p < 0.01, ***p < 0.001

To identify candidate molecules that preferentially inhibit β-catenin-activated tumors and could be readily translated into clinical practice, we first deleted exon 3, the most altered site in *CTNNB1* gene, to establish constitutive β-catenin-activated MEFs (*β-catenin*^*Δ(ex3)/*+^) [23]. We then conducted a high-throughput screening with a chemical library containing 1088 FDA-approved drugs for molecules that selectively inhibited the β-catenin-activated cells but not WT MEFs (Fig. 1A). Among the top hits that preferentially suppressed β-catenin-activated cells, iron chelator DFX was chosen for validation. DFX specifically decreased *β-catenin*^*Δ(ex3)/*+^ cell viability (Fig. 1B). We confirmed the efficacy of iron chelating with other two iron chelators, DFO and VLX600. Consistently, both DFO and VLX600 preferentially reduced *β-catenin*^*Δ(ex3)/*+^ MEF viability (Fig. 1C). In addition, iron supplementation with FAC rescued DFO, DFX and VLX600-mediated cell proliferation arrest (Fig. 1D). Therefore, activated β-catenin cells are sensitive to iron depletion.

### Depleting iron suppresses β-catenin-activated tumor growth

**Fig. 2:**
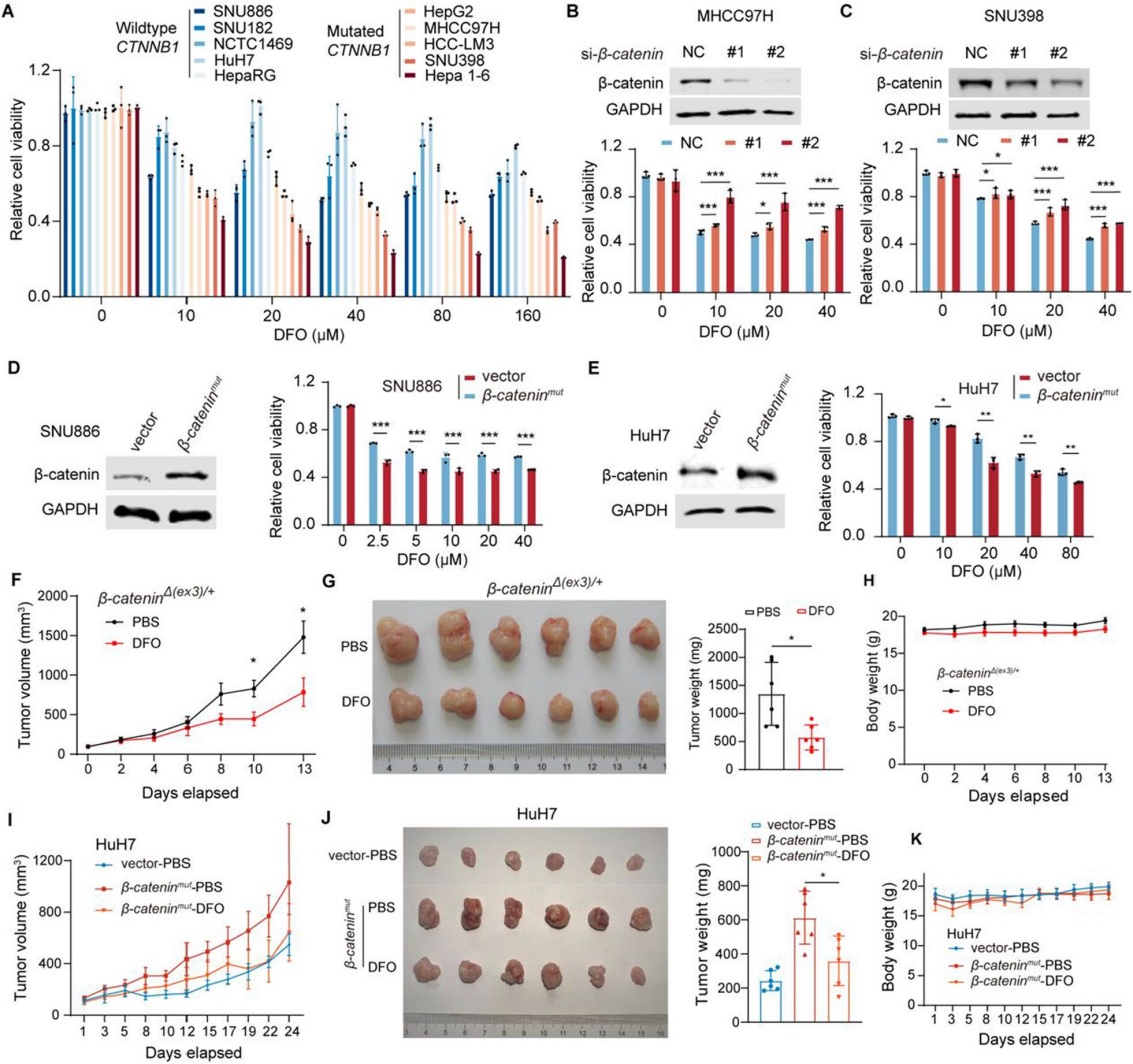
DFO preferentially suppresses β-catenin-activated cell proliferation and tumor formation. **A**–**E**, Viability of cells treated with DFO at different concentrations for 48 h. 10 hepatic cell lines (**A**); MHCC97H (B) or SNU398 (**C**) transfected with control or *β-catenin* siRNA; and SNU886 (**D**) or HuH7 (**E**) transfected with vector or *β-catenin*^*mut*^ plasmid. Protein abundances were analyzed (**B**, up; **C**, up; **D**, left; **E**, left). **F**–**H,** Nude mice subcutaneously inoculated with *β-catenin*^*Δ(ex3)/*+^ MEFs were administered intraperitoneally with PBS or DFO (400 mg/kg) every other day. Tumor growth was calculated as the mean value in tumor volume (**F**). Tumor images, tumor weights (**G**), and body weights (**H**) were plotted at the end of treatment;* n* = 6. **I**–**K,** Nude mice were subcutaneously inoculated with HuH7 transfected with vector or *β-catenin*^*mut*^ plasmid. These mice were administered intraperitoneally with PBS or DFO (400 mg/kg) every other day. Tumor growth was calculated as the mean value in tumor volumes (**I**). Tumor images, tumor weights (**J**), and body weights (**K**) were plotted at the end of treatment;* n* = 6. Data were shown as mean ± SD and analysis was performed using *t* test. *p < 0.05, ***p < 0.001

To investigate the efficacy of iron deprivation in liver cancer treatment, DFO was chosen for further study as it less likely causes rashes, gastrointestinal symptoms, and severe adverse events than DFX does [28]. We treated 10 hepatic cell lines which consisted of 2 mouse-derived (1 immortalized, 1 malignant) and 8 human-derived (1 immortalized, 7 malignant) liver cell lines (Additional file 1: Table S1). β-catenin mutant cell lines were more sensitive to DFO than WT ones (Fig. 2A). Knocking down β-catenin compromised the sensitivity of *CTNNB1*-mutated MHCC97H and SNU398 cells to DFO treatment (Fig. 2B, C). In contrast, overexpression of oncogenic *β-catenin*^*mut*^ which harbored S33A, S37A, T41A and S45A mutations sensitized SNU886 and HuH7 cells to DFO treatment (Fig. 2D, E). Cellular sensitivity to DFO is thus positively correlated with β-catenin activity. DFO inhibited tumorigenicity of *β-catenin*^*Δ(ex3)/*+^ MEFs, manifesting as reduced tumor volumes and tumor weights, with negligible impact on body weights in nude mice (Fig. 2F–H). HuH7 cells transfected with oncogenic *β-catenin*^*mut*^ were more tumorigenic than the cells transfected with vector alone in nude mice. The tumorigenesis of oncogenic β-catenin-expressed cells was blunted by DFO treatment (Fig. 2I, J), while DFO had marginal impact on body weights of treated mice (Fig. 2K). Collectively, iron chelator repressed β-catenin-activated tumor formation.

Since iron plays multifaceted roles in cancer biology, addition of excessive iron and induction of iron-dependent ferroptosis have been reported to treat cancer [29, 30]. To check whether β-catenin-activated cells were liable to these interventions, we treated MEFs with iron supplements or ferroptosis inducer. Supplementation of FeCl_2_ or FAC didn’t alter cell viability of both WT and *β-catenin*^*Δ(ex3)/*+^ MEFs (Additional file 1: Fig. S1A, B). *β-catenin*^*Δ(ex3)/*+^ MEFs were more resistant to erastin-induced ferroptosis (Additional file 1: Fig. S2), which was consistent with glutathione peroxidase 4-mediated ferroptosis resistance in β-catenin-activated cancers [31]. These data suggest that iron chelation but not iron addition and ferroptosis induction is promising for β-catenin-activated cancer treatment.

### β-catenin increases cellular labile iron pool

**Fig. 3:**
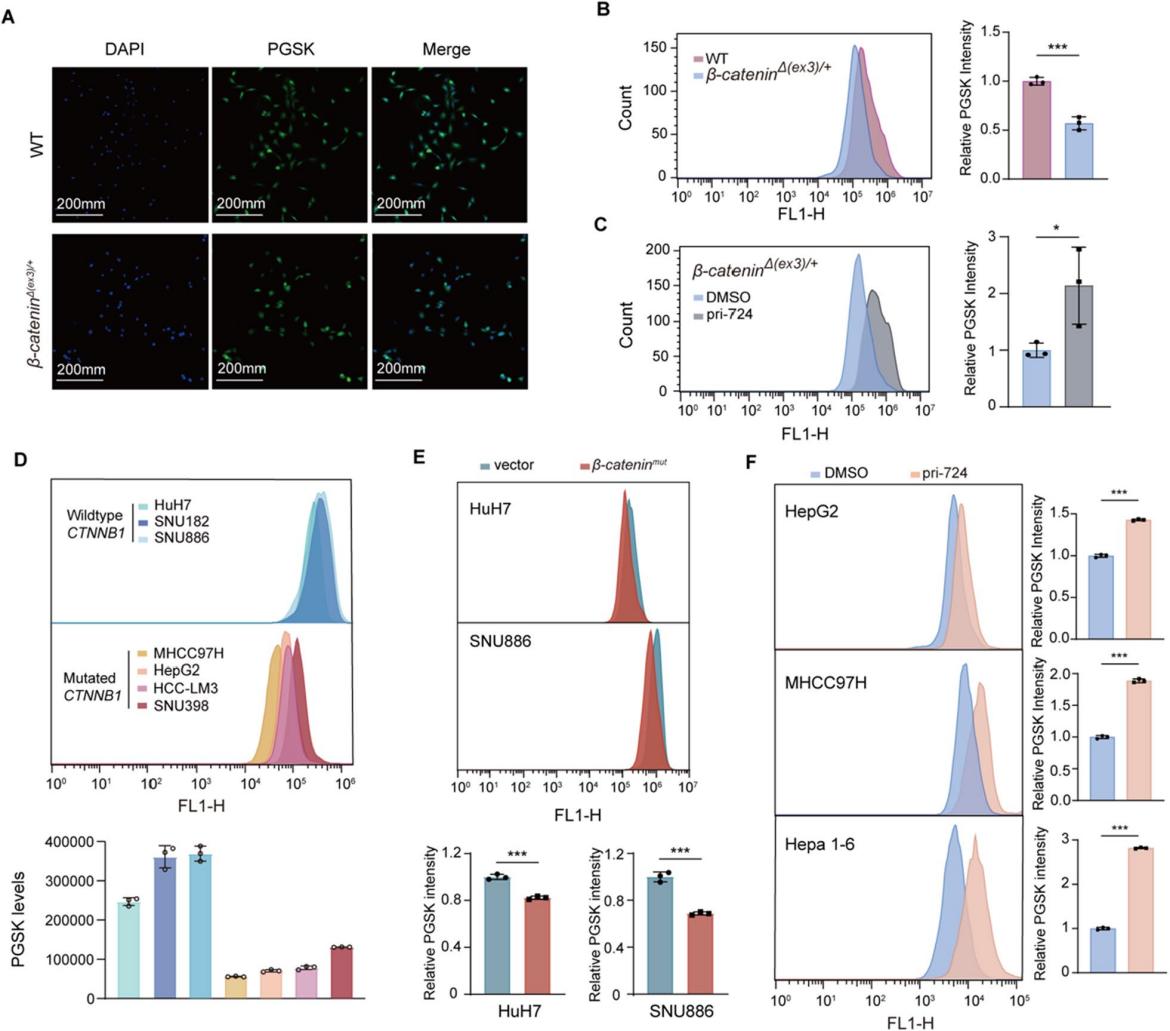
Oncogenic β-catenin elevates cellular LIP. **A**, Representative confocal images of WT and *β-catenin*^*Δ(ex3)/*+^ MEFs using iron-sensitive fluorescent PGSK dye. **B**–**F,** Flow cytometry analysis of PGSK intensity in WT and *β-catenin*^*Δ(ex3)/*+^ MEFs (**B**); *β-catenin*^*Δ(ex3)/*+^ MEFs treated with DMSO or 20 μM pri-724 for 48 h (**C**); 7 hepatic cancer cell lines (**D**); HuH7 and SNU886 transfected with vector or *β-catenin*^*mut*^ plasmid (**E**); HepG2, MHCC97H and Hepa 1–6 treated with DMSO or 20 μM pri-724 for 48 h (**F**);* n* = 3. Data were shown as mean ± SD and analysis was performed using *t* test. *p < 0.05, ***p < 0.001

Either addiction to excessive iron or clinging to limited iron might render *CTNNB1* mutant cells susceptible to iron chelators. We checked chelatable or LIP abundance in *CTNNB1* mutant cells with a fluorescent indicator PGSK. PGSK fluorescence is quenched upon interaction with LIP. Compared to WT MEFs, *β-catenin*^*Δ(ex3)/*+^ MEFs had higher LIP (Fig. 3A, B). β-catenin inhibitor pri-724 reduced LIP in *β-catenin*^*Δ(ex3)/*+^ MEFs (Fig. 3C). More LIP was detected in β-catenin-activated liver cancer cells than in the WT ones (Fig. 3D). Oncogenic β-catenin overexpression enhanced cellular LIP in *CTNNB1* WT cells (Fig. 3E). Moreover, pri-724 decreased LIP in *CTNNB1*-mutated HepG2, HCCM97H and Hepa 1–6 cells (Fig. 3F). All these data indicate that β-catenin raises intracellular LIP.

### β-catenin stimulates IRP2 transcription to increase cellular LIP

**Fig. 4:**
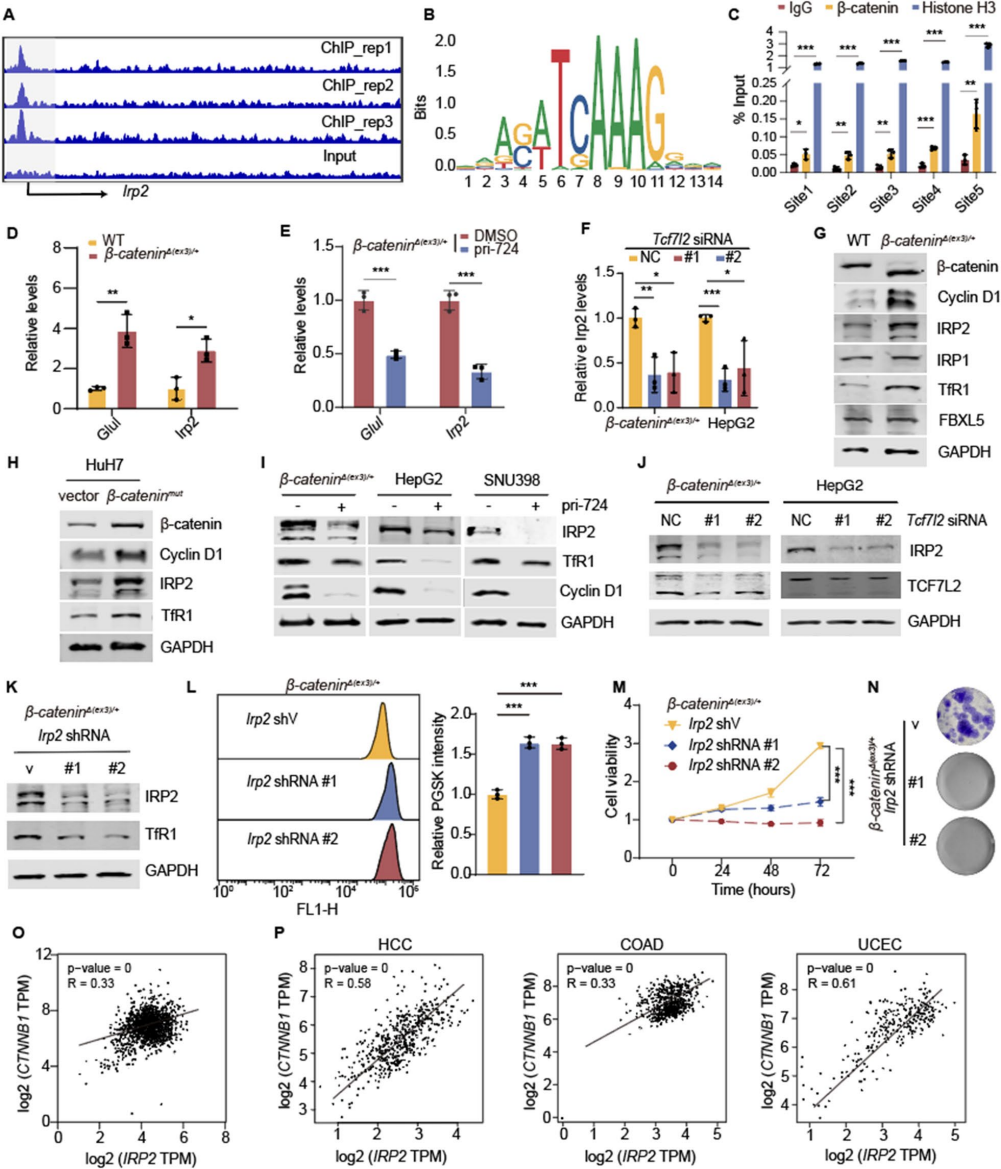
β-catenin transcriptionally stimulates IRP2 to boost LIP and cell proliferation. **A,** Genome browser track of β-catenin ChIP-seq profiles at Irp2 loci. **B,** TCF7L2 binding motif analysis using JASPAR. **C,** β-catenin antibody-precipitated DNA was PCR amplified for *Irp2* promoter regions; n = 3. **D**–**F,** *Irp2* mRNA levels in WT and *β-catenin*^*Δ(ex3)/*+^ MEFs (**D**), *β-catenin*^*Δ(ex3)/*+^ MEFs treated with DMSO or 20 μM pri-724 for 48 h (**E**), and *β-catenin*^*Δ(ex3)/*+^ MEFs or HepG2 cells transfected with control or *Tcf7l2* siRNA (F); *n* = 3. **G**–**J,** Immunoblotting of WT and *β-catenin*^*Δ(ex3)/*+^ MEFs (**G**); HuH7 transfected with vector or *β-catenin*^*mut*^ plasmid (**H**); *β-catenin*^*Δ(ex3)/*+^ MEFs, HepG2 and SNU398 treated with DMSO or 20 μM pri-724 for 48 h (**I**); *β-catenin*^*Δ(ex3)/*+^ MEFs and HepG2 cells transfected with control or *Tcf7l2* siRNA (**J**). **K**–**N,** *β-catenin*^*Δ(ex3)/*+^ MEFs were transfected with control or *Irp2* shRNA. Analysis of IRP2 abundance (**K**), cellular PGSK intensity (**L**), cell proliferation (**M**), and colony formation (**N**) were performed; *n* = 3. **O**, **P,** Correlation between *CTNNB1* and *IRP2* mRNA abundance. Data of cancer cell lines were generated from DepMap database (**O**). Data of HCC, COAD and UCEC were from GEPIA database (**P**). Data were shown as mean ± SD and analysis was performed using *t* test. *p < 0.05, **p < 0.01, ***p < 0.001

Β-catenin is a transcriptional cofactor that binds transcription factor 7 like 2 (TCF7L2, also known as TCF4) to drive target gene expression [1]. We therefore analyzed β-catenin ChIP-seq data to dissect the molecular mechanism responsible for *β*-catenin-boosted LIP [23]. β-catenin was enriched in the promoter region of *Irp2* gene (Fig. 4A), which is critical for cellular iron homeostasis via post-transcriptional stabilization of transferrin receptor 1 (TfR1, TFRC) mRNA [29]. JASPAR database analysis revealed the TCF7L2 binding motif, which is conserved between human and mouse, in *IRP2* gene (Fig. 4B) [27, 32]. ChIP-PCR assay indeed detected the interaction of β-catenin with *Irp2* promoter regions (Fig. 4C). Moreover, β-catenin-activated cells had more *Irp2* mRNA (Fig. 4D), which was attenuated by pri-724 treatment or *Tcf7l2* knockdown (Fig. 4E, F). IRP2 and TfR1 proteins were enriched in β-catenin-activated cells (Fig. 4G, H) and decreased in pri-724-treated cells (Fig. 4I). TCF7L2 silence decreased IRP2 protein levels in *β-catenin*^*Δ(ex3)/*+^ MEFs and HepG2 (Fig. 4J). Therefore, β-catenin may bind to TCF7L2 to stimulates IRP2 transcription. Unlike IRP2, IRP1 proteins was not changed in WT and *β-catenin*^*Δ(ex3)/*+^ MEFs (Fig. 4G). As an iron sensor, F-box/LRR-repeat protein 5 (FBXL5) is degraded in low-iron conditions caused by DFO [33]. Suppression of FBXL5 decreases ubiquitylation and degradation of IRP2 proteins [34]. Since the protein abundances of FBXL5 did not differ between WT and *β-catenin*^*Δ(ex3)/*+^ MEFs (Fig. 4G), β-catenin increased IRP2 through TCF7L2-mediated transcription activation but not inhibition of FBXL5-mediated degradation.

To elucidate the significance of IRP2 in β-catenin-activated cells, we silenced *Irp2* in *β-catenin*^*Δ(ex3)/*+^ MEFs (Fig. 4K). IRP2 knockdown dropped cellular LIP levels (Fig. 4L) and weakened cell viabilities (Fig. 4M, N). In addition, we validated the relationship between IRP2-TfR1 axis and β-catenin activity in human cancers. β-catenin mRNA was positively correlated with mRNAs of IRP2 and TfR1 in 1406 cancer cell lines (Fig. 4O, Additional file 1: Fig. S3A) and β-catenin-frequently activated human HCC, COAD and UCEC samples (Fig. 4P, Additional file 1: Fig. S3B). WNT/β-catenin-altered tumors contained more IRP2 and TfR1 than WNT/β-catenin-unaltered tumors (Additional file 1: Fig. S3C, D). β-catenin is thus positively correlated with IRP2-TfR1 signaling cascade in human cancers. Taken together, β-catenin-activated tumors potentiate *Irp2* transcription to enhance cellular LIP levels and cell proliferation.

### Iron depletion impairs mitochondrial function to suppress β-catenin-mediated tumor formation

**Fig. 5:**
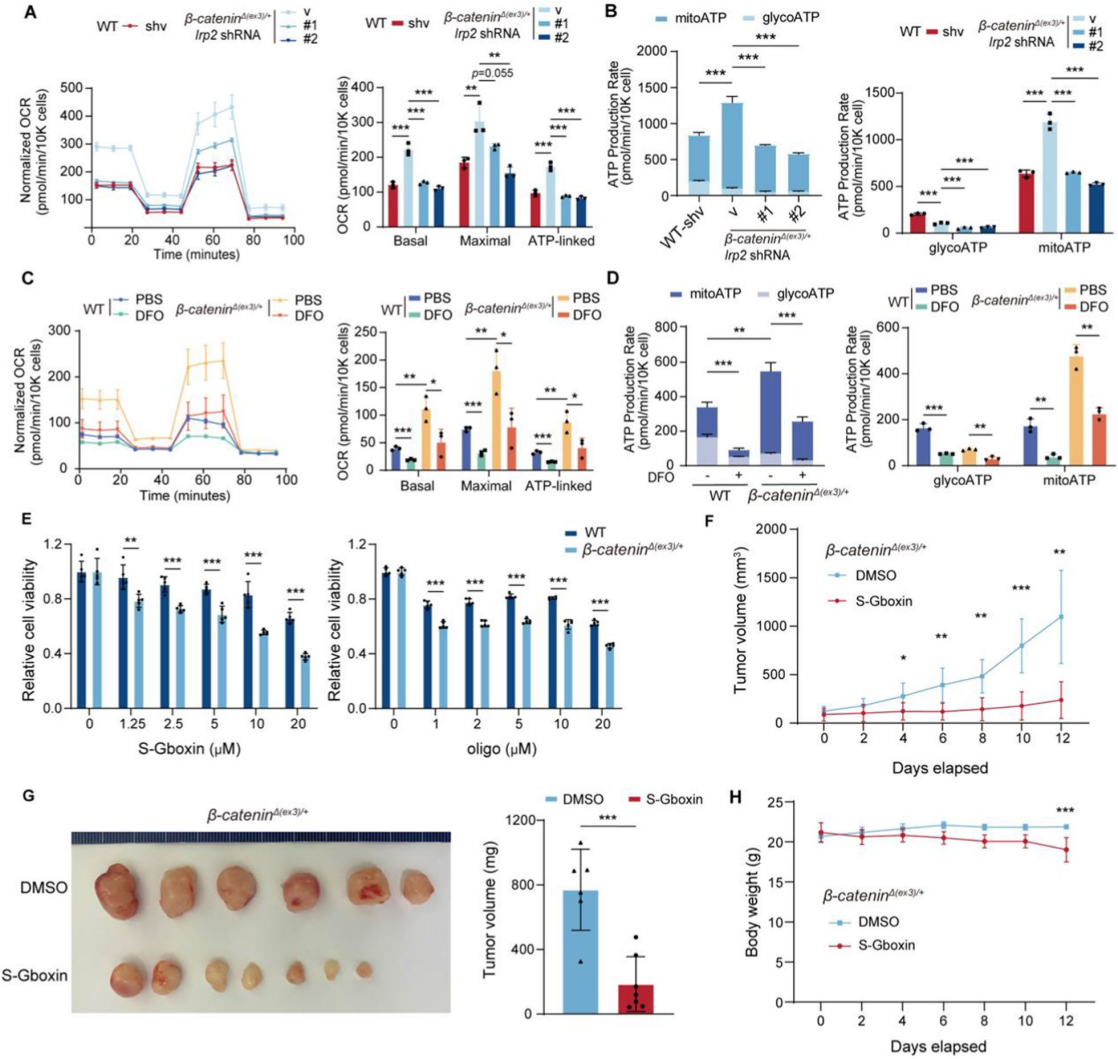
Iron deprivation blunts β-catenin-activated tumor formation via impairing mitochondrial function **A**, **B,** oxygen consumption rate (OCR) (**A**, left) and ATP production rate (**B**, left) in *β-catenin*^*Δ(ex3)/*+^ MEFs transfected with control or IRP2 shRNA. OCR for basal and maximal respiration (**A**, right); Glycolytic and mitochondrial ATP production rates (**B**, right); n = 3. **C**, **D,** OCR (**C**, left) and ATP production rate (**D**, left) in WT and *β-catenin*^*Δ(ex3)/*+^ MEFs treated with control or 5 μM DFO for 48 h. OCR for basal and maximal respiration (C, right); Glycolytic and mitochondrial ATP production rates (D, right); n = 3. **E,** Viability of WT and *β-catenin*^*Δ(ex3)/*+^ MEFs treated with S-Gboxin or oligomycin A at different concentrations for 48 h; *n* = 5. **F**–**H,** Nude mice subcutaneously inoculated with *β-catenin*^*Δ(ex3)/*+^ MEFs were treated with PBS (n = 6) or S-Gboxin (10 mg/kg) every other day, n = 7. Tumor growth was calculated as the mean value in tumor volumes (**F**). Tumor images, tumor weights (**G**), and body weights (**H**) were plotted at the end of treatment. Data were shown as mean ± SD and analysis was performed using *t* test. *p < 0.05, **p < 0.01, ***p < 0.001

Because iron-sulfur mediates electron transfer in mitochondrial respiratory chain, iron plays critical roles in maintenance of mitochondrial function [35]. Mitochondrial activity is enhanced in β-catenin-activated cancer to fuel tumorigenesis [5]. IRP2 knockdown dropped cellular OCR levels (Fig. 5A) and mitochondrial ATP production (Fig. 5B) in these cells. DFO also reduced OCR (Fig. 5C), mitochondrial ATP production and total ATP production (Fig. 5D) of *β-catenin*^*Δ(ex3)/*+^ MEFs. Similar to iron chelators, direct suppression of mitochondrial metabolism with oligomycin (mitochondrial complex I inhibitor) or S-Gboxin (ATP synthetase inhibitor) preferentially hindered *β-catenin*^*Δ(ex3)/*+^ MEF proliferation (Fig. 5E). In addition, S-Gboxin suppressed tumorigenicity of *β-catenin*^*Δ(ex3)/*+^ MEFs with minimal impact on mouse body weights (Fig. 5F–H), suggesting iron-mediated mitochondrial metabolism is a targetable vulnerability for β-catenin activated tumors.

## Discussion

**Fig. 6:**
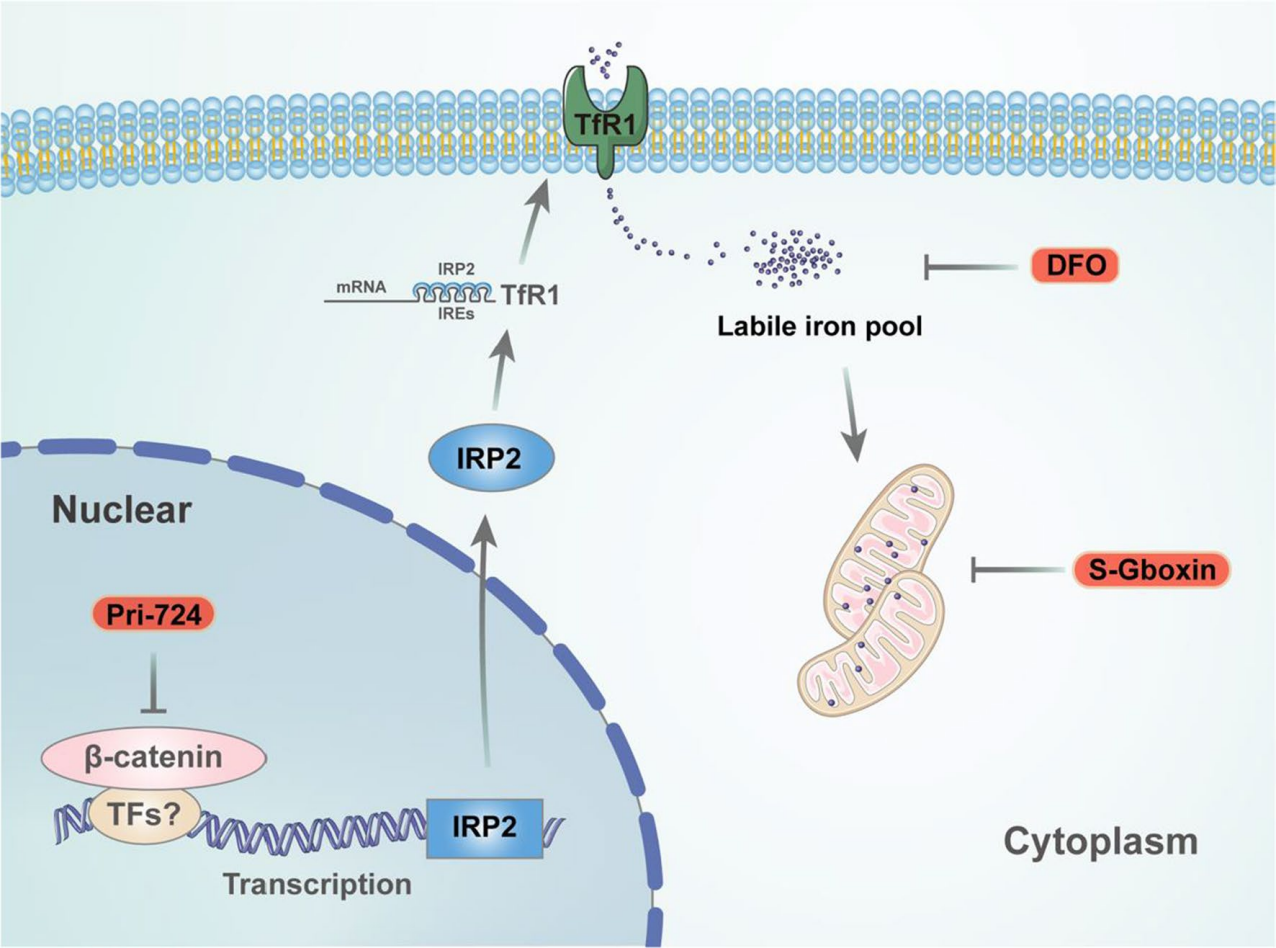
Illustration of β-catenin-IRP2-iron stimulation of mitochondrial metabolism promotes cell proliferation

Β-catenin signaling pathway plays important roles in regulation of cell growth and proliferation. Gain-of-function mutations of β-catenin are common in human cancers. In this study, we found that oncogenic β-catenin augmented intracellular iron to promote cell proliferation and tumorigenesis. Mechanistically, β-catenin transcriptionally stimulated IRP2 expression to enhance cellular LIP which contributed to the enhanced mitochondrial function. The boosted IRP2-iron-mitochondrial function is druggable for β-catenin-activated tumors (Fig. 6).

No approved target therapy is available for β-catenin-activated tumor. Repurposing existing drugs, with the advantages of lower risk, shorter timelines, and less investment, expedites drug discovery and development. For example, Paxlovid is quickly approved for COVID-19 treatment [36]. Through screening of 1088 approved drugs, we discovered that iron chelators selectively suppressed β-catenin-activated cell viability. The first approved iron chelator for iron-overload-related disease, DFO, with higher safety and lower price than DFX [37], deterred β-catenin-activated tumorigenesis.

DFO exhibits anticancer effect in breast cancer cell lines and lung cancer cell-derived xenograft tumor [38, 39]. In combination with cytosine arabinoside, DFO causes striking cytoreduction in a patient with acute leukemia [40]. However, limited activity (20% overall response rate) was observed in 10 DFO-treated patients with treatment-refractory metastatic HCC [41]. 70% hepatic cancer without *CTNNB1* mutation may contribute to the low response rate of genetically unclassified HCC to DFO [14, 15].

It is known that iron activates Wnt/β-catenin signaling [42–45]. We found elevated chelatable iron in β-catenin-activated cells. A potential positive feedback loop between β-catenin activation and iron overload may contribute to tumor development. Iron overload is associated with increased risk for cancers, particularly gastrointestinal cancers and HCC [46–48]. Excess iron accelerates HCC and CRC development in mice, whereas iron deprivation blocks myeloma cell growth and mouse CRC development [49–52]. In patients with chronic hepatitis C, long-term low-iron diet decreases the risk of HCC development [53]. Because iron chelation selectively reduced β-catenin-activated cell viability and tumor formation, we suggest that increased LIP is necessary in oncogenic β-catenin-induced cancer development.

β-catenin promotes stabilization and nuclear translocation of hypoxia inducible factor 1α (HIF-1α) [55]. Nuclear β-catenin cooperates with HIF-1α at the promoter regions of HIF-1α target genes to enhance HIF-1α-mediated transcription [56]. In addition, HIF-1α stimulates cellular iron uptake by inducing TfR1 expression [57–60]. Therefore, β-catenin enhancement of HIF-1α may contribute to β-catenin-stimulated iron accumulation we identified in this study. Intracellular iron homeostasis is strictly secured by IRP1/2 [54]. We demonstrated that transcriptional activation of IRP2 by β-catenin also contributed to the elevated LIP. Despite the well-established function of IRP2 in securing iron metabolism, little is known about its role in cancer development. We found that IRP2 knockdown not only decreased LIP but also blocked proliferation of β-catenin-activated cells.

Iron plays various roles such as mitochondrial metabolism, DNA synthesis, cell cycle progression and redox homeostasis, deprivation of iron may cause various pathological changes [29]. Iron chelating causes redox damage, cell cycle arrest, and DNA replication impairment to repress cell proliferation and tumorigenesis [50, 61–63]. We demonstrated that iron deprivation impaired β-catenin-invigorated mitochondrial activity. Therefore, iron chelators may play anti-tumor roles through multiple mechanisms in addition to mitochondrial dysfunction we presented here. Mitochondria bestow flexibility on tumor cells to adapt to cellular and environmental alterations [54]. Unlike most tumors that use the Warburg effect, β-catenin-activated tumors are addicted to mitochondria-supplied energy [5]. In this study, pharmaceutical inhibition of mitochondrial function by S-Gboxin hindered oncogenic β-catenin-mediated cell viability and tumor development. Thus, suppressing mitochondrial function is a promising therapeutic strategy for β-catenin-activated tumors.

## Conclusions

Β-catenin-activated cells exhibit an augmented dependency on iron availability. Oncogenic β-catenin transactivates IRP2 to enhance mitochondrial function via raising intracellular LIP. Β-catenin/IRP2/iron-boosted mitochondrial energetics is essential for cell proliferation and tumor growth. Intervention of iron, IRP2 or mitochondrial function abolishes β-catenin-activated cancers. Since DFO is an old drug, its efficacy can be readily tested for the treatment of oncogenic β-catenin-related tumors.

## Supplementary Information

**Additional file 1: Figure S1.** Iron addition has no impact on cell proliferation of WT and *β-catenin*^*Δ(ex3)/*^^+^ MEFs. **Figure S2.** β-catenin-activated MEFs are resistant to ferroptosis induction. **Figure S3.** β-catenin is positively correlated with IRP2 and TfR1. **Table S1.** Mutations of cell lines.

## Data Availability

The datasets supporting the conclusions of this article are included within the article and its additional files.

## Abbreviations

AKT2: AKT Serine/Threonine Kinase 2
APC: Adenomatous polyposis coli
CBP: Cyclic adenosine monophosphate response element binding protein-binding protein
COAD: Colon adenocarcinoma
CRC: Colorectal cancer
CCK8: Cell Counting Kit-8
DFO: Deferoxamine mesylate
DFX: Deferasirox
FAC: Ferric ammonium citrate
FAO: Fatty acid oxidation
FBXL5: F-box/LRR-repeat protein 5
FGF: Fibroblast growth factor
GLUL: Glutamate-ammonia ligase
HCC: Hepatocellular carcinoma
HIF-1α: Hypoxia-inducible factor-1α
IRP2: Iron-regulatory protein 2
LIP: Labile iron pool
MEFs: Mouse embryonic fibroblasts
OCR: Oxygen consumption rate
PFA: Paraformaldehyde
PGSK: Phen Green™ SK
PK: Pharmacokinetics
PPAP: Peroxisome proliferator activated receptor alpha
TCF: T cell factor
TCF7L2: Transcription factor 7 like 2
TERT: Telomerase reverse transcriptase
UCEC: Uterine corpus endometrial carcinoma
WT: Wildtype
