# Supplementary material for: β-catenin-IRP2-primed iron availability to mitochondrial metabolism is druggable for active β-catenin-mediated cancer
Source: J Transl Med. 2023 Jan 26;21:50. doi: 10.1186/s12967-023-03914-0 (PMC9879242; doi:10.1186/s12967-023-03914-0)
Supplement: Supplementary file 1 — Additional file 1: Figure S1. Iron addition has no impact on cell proliferation of WT and β-cateninΔ(ex3)/+ MEFs. Figure S2. β-catenin-activated MEFs are resistant to ferroptosis induction. Figure S3. β-catenin is positively correlated with IRP2 and TfR1. Table S1. Mutations of cell lines. [file 12967_2023_3914_MOESM1_ESM.docx]

Supplementary Data for

**β-catenin-IRP2-primed iron availability to mitochondrial metabolism is druggable for active β-catenin-mediated cancer.**

Yuting Wu^1#^, Shuhui Yang^1#^, Luyang Han^1^, Kezhuo Shang^1^, Baohui Zhang^2^, Xiaochen Gai^1^, Weiwei Deng^1^, Fangming Liu^1*^, Hongbing Zhang^1*^.

*#Equal author contribution*

^1^State Key Laboratory of Medical Molecular Biology, Haihe Laboratory of Cell Ecosystem, Department of Physiology, Institute of Basic Medical Sciences and School of Basic Medicine, Chinese Academy of Medical Sciences and Peking Union Medical College, Beijing, China.

^2^Department of Physiology, School of Life Science, China Medical University, Shenyang, China.

**Corresponding author:**

Fangming Liu

Email address: lfmpumc@163.com

Mailing address: 5 Dong Dan San Tiao, Institute of Basic Medical Sciences, Beijing, China.

Hongbing Zhang

Email address: hbzhang@ibms.pumc.edu.cn

Mailing address: 5 Dong Dan San Tiao, Institute of Basic Medical Sciences, Beijing, China.

This document includes:

Supplementary Figures S1 to S3 and Figure legends.

Supplementary Table S1.

**Supplementary Figures**

**
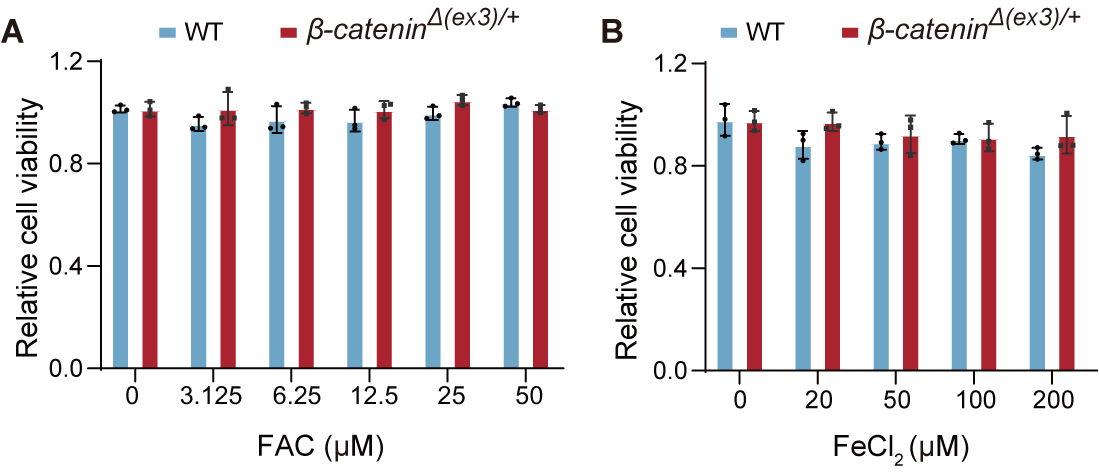
**

**Supplementary Figure S1. Iron addition has no impact on cell proliferation of WT and *β-catenin^Δ(ex3)/^*^+^ MEFs.**

Cell viability of WT and *β-catenin^Δ(ex3)/^*^+^ MEFs treated with FAC (A) or FeCl_2_ (B) at different concentrations for 48 hours; n=3. Data were shown as mean ± SD and analysis was performed using *t* test.


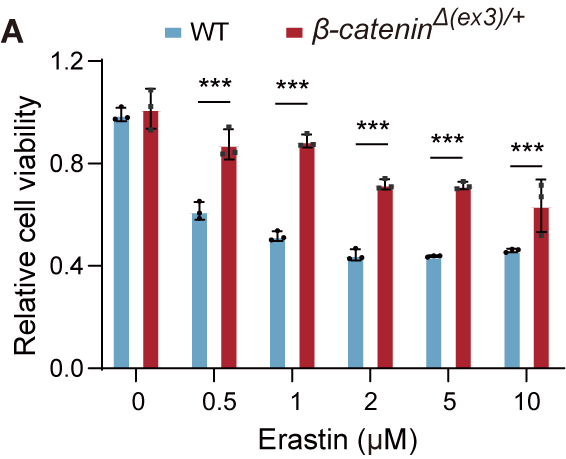


**Supplementary Figure S2. β-catenin-activated MEFs are resistant to ferroptosis induction.**

Cell viability of WT and *β-catenin^Δ(ex3)/^*^+^ MEFs treated with erastin at different concentrations for 48 hours; n=3. Data were shown as mean ± SD and analysis was performed using *t* test. ****p* < 0.001.


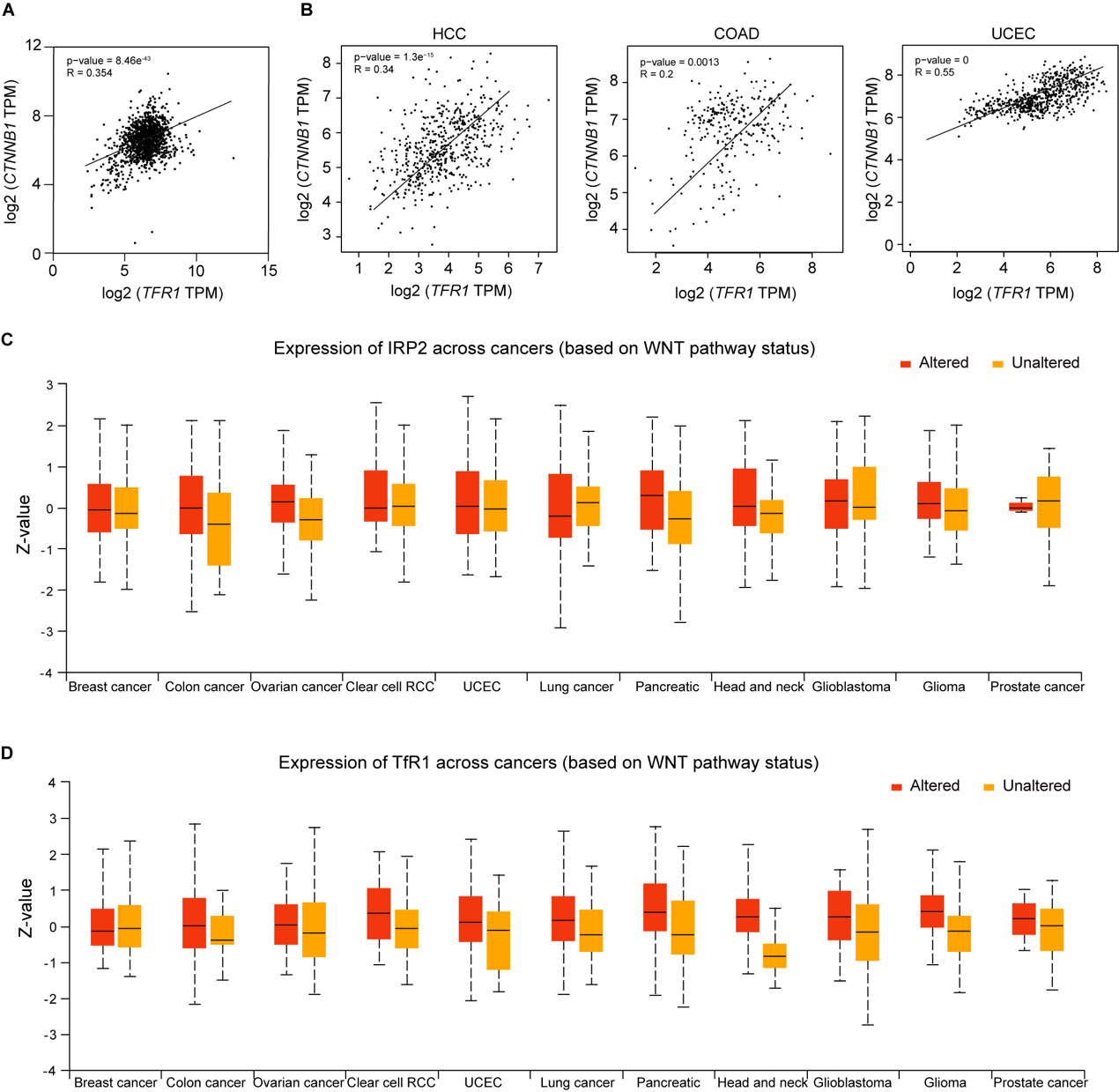


**Supplementary Figure S3. β-catenin is positively correlated with IRP2 and TfR1.**

**A and B,** Correlation between *CTNNB1* and *TFRC* mRNA abundance. Data of cancer cell lines were generated from DepMap database (A). Data of HCC, COAD and UCEC were from GEPIA database (B). **C and D,** Protein analyses of IRP2 (C) or TfR1 (D) across different cancers with altered or unaltered WNT pathway were conducted on UALCAN database.

| **Cell Line** | **Species** | **Disease** | **Mutation** | | | | **Reported in** |  | |
| --- | --- | --- | --- | --- | --- | --- | --- | --- | --- |
|  |  |  | ***CTNNB1*** | ***APC*** | ***AXIN2*** | ***AXIN1*** |  |  | |
| HepaRG | *Homo sapiens* | Normal | no | no | no | no | [1] |  | |
| Huh7 | *Homo sapiens* | HCC | no | no | no | no | [2] |  | |
| SNU182 | *Homo sapiens* | HCC | no | no | no | no | [2] |  | |
| SNU886 | *Homo sapiens* | HCC | no | no | no | no | [2] |  | |
| NCTC1469 | *Mus musculus* | Normal | no | no | no | no | [3] |  | |
| MHCC97H | *Homo sapiens* | HCC | YES | no | no | no | [2] |  | |
| SNU398 | *Homo sapiens* | HCC | YES | no | no | no | [2] |  | |
| Hepa 1-6 | *Mus musculus* | Hepatoma | YES | unknown | unknown | unknown | [4] | |  |
| HepG2 | *Homo sapiens* | Hepatoblastoma | YES | no | no | no | [2] |  | |
| HCCLM3 | *Homo sapiens* | HCC with high metastatic potential | YES | unknown | unknown | unknown | [5] |  | |

**Supplementary table S1. Mutations of cell lines**

**References**

1. Yang H, Sun L, Pang Y, Hu D, Xu H, Mao S, Peng W, Wang Y, Xu Y, Zheng YC, et al: **Three-dimensional bioprinted hepatorganoids prolong survival of mice with liver failure.** *Gut* 2021, **70:**567-574.

2. **Liver Cancer Cell Lines Database** [<https://lccl.zucmanlab.com/hcc/cellLines>]

3. **ATCC: The Global Bioresource Center** [<https://www.atcc.org/products/ccl-9.1>]

4. Riou R, Ladli M, Gerbal-Chaloin S, Bossard P, Gougelet A, Godard C, Loesch R, Lagoutte I, Lager F, Calderaro J, et al: **ARID1A loss in adult hepatocytes activates beta-catenin-mediated erythropoietin transcription.** *Elife* 2020, **9**.

5. Li Y, Tang Y, Ye L, Liu B, Liu K, Chen J, Xue Q: **Establishment of a hepatocellular carcinoma cell line with unique metastatic characteristics through in vivo selection and screening for metastasis-related genes through cDNA microarray.** *J Cancer Res Clin Oncol* 2003, **129:**43-51.
